# Improving Foaming Properties and Quality of Pasteurized Milk Using Antimicrobial Agents from Wild *Pediococcus acidilactici*

**DOI:** 10.3390/foods14040641

**Published:** 2025-02-14

**Authors:** Sameh Awad, Abeer I. M. EL-Sayed, Dina Amer, Dalia Atef, Mona Ashraf, Jianquan Kan, Muying Du, Khaled Elsaadany

**Affiliations:** 1 Department of Dairy Science and Technology, Faculty of Agriculture, Alexandria University, Alexandria 21526, Egypt; daliaatef390@gmail.com (D.A.); monaasharf@gmail.com (M.A.); elsaadany12@hotmail.com (K.E.); 2 Botany and Microbiology Department, Faculty of Science, Damanhour University, Damanhour 22511, Egypt; abeer.elsayed@sci.dmu.edu.eg; 3 Department of Food and Dairy Science and Technology, Faculty of Agriculture, Tanta University, Tanta 31527, Egypt; 4 College of Food Science, Southwest University, Chongqing 400715, China; ganjq1965@163.com (J.K.); muyingdu@swu.edu.cn (M.D.); 5 Chinese-Hungarian Cooperative Research Center for Food Science, Southwest University, Chongqing 400715, China; 6 Chongqing Key Laboratory of Specialty Food Co-Built by Sichuan and Chongqing, Chongqing 400715, China

**Keywords:** foam milk, free fatty acids, bacteriocin, wild *Pediococcus acidilactici*

## Abstract

Pasteurized milk foam has become a quality issue in some applications, such as cappuccino-style drinks, as it should be stable and high-capacity. The extended shelf life of pasteurized milk is also a challenge. Some factors affect the foam capacity and stability; among them, the increasing amount of free fatty acids in raw milk is critical. The psychrotrophic bacteria can produce a lipase-like enzyme, which is responsible for increasing the level of free fatty acids in raw milk. Therefore, this work aims to utilize the cell-free supernatant of a bacteriocin-producing culture as a natural preservative against psychrotrophic and spore-forming bacteria to enhance the foaming capacity and stability and improve the final product’s quality and shelf life. Milk samples from 15 dairy farms were assessed for free fatty acids, microbiological quality, and foaming capacity. Raw milk was divided into four portions: a control without any additive and cell-free supernatant (CFS) treatments, with CFS added at concentrations of 5, 10, and 15 mL/L in each portion. Raw milk was stored for 5 days before heat treatment at 75 °C/30 s, then cooled at 5 °C. All samples were examined for microbiological, free fatty acid, and foaming properties immediately after heat treatment and during storage up to 14 days. The results of this study reveal that there is a negative impact of free fatty acids on the capacity and stability of foaming. The cell-free supernatant (15 mL/L) of the traditional dairy isolate *Pediococcus acidilactici* inhibits the psychrotrophic bacteria in raw milk during storage for 5 days, a phenomenon which has a direct impact on reducing the free fatty acids, improving the foaming capacity and stability, as well as reducing the bitterness at the end of the shelf life of pasteurized milk up to 14 days compared to the detection of bitterness after 8 days in the control pasteurized milk. It is concluded that, to produce pasteurized milk with a high foaming capacity and extended shelf life, raw milk with low amounts of free fatty acids should be used and fast pasteurized or treated with a bacteriocin of lactic acid bacteria.

## 1. Introduction

Cappuccino-style drinks have become more popular in recent years. The main selling feature of these products is the foam layer on top, which greatly enhances their mouthfeel, texture, and appearance, all of which reflect quality, affected by different factors like its composition, processing, and additives. Furthermore, the quality of milk foam changes throughout the year depending on the season. The seasonal variation in the quality of milk foam is related to variations such as fat and protein fractions. Seasonality influences not just the amount of protein in milk, but also the proportions of milk proteins and the ratio of casein to whey protein [1,2].

Lipase activity from different sources in dairy products releases free fatty acids (FFAs). The lipases in butter are endogenous lipases found in raw milk [3] and in enzymes of starter and non-starter bacteria. Psychrotrophic bacteria have a major effect on the level of free fatty acids (FFAs) in raw milk during cold storage and transferring because they produce heat-stable lipases, which hydrolyze lipids into FFAs [4]. This lowers the quality and shelf life of milk and dairy products by causing rancid flavors, off-tastes, and milk spoiling. Handling, cleanliness, and avoiding storing raw milk for extended periods before heat treatments are essential in the manufacturing and processing of milk to reduce these effects [4,5].

Protein films that develop around air bubbles during foaming are prone to becoming unstable due to the presence of free fatty acids. The main factor preventing air bubbles from popping is milk proteins [6]. FFAs, however, have the potential to interfere with these proteins’ surface-active characteristics, decreasing their capability to form stable films and, consequently, their foaming capacity [7]. FFAs can form a layer around air bubbles, upsetting the protein network and impeding the development of foam. As a result, milk’s capacity to hold onto air is diminished, which might cause poor foam production or foam that collapses quickly once it is created [8,9].

Milk fat can limit the milk’s ability to foam due to the fat destabilizing the air bubbles in the milk. The emulsification of fat is enhanced by the presence of FFAs, further impeding the capacity of milk to foam. By stabilizing fat globules and acting as emulsifiers, FFAs further reduce foam formation by preventing proteins from fully participating in the foaming process [10]. Rapid foam disintegration is a result of the high FFA content in raw milk. FFAs promote the production of bigger air bubbles that are more likely to collapse and cluster. This implies that, even if the foam is generated, it will not remain stable for a long time before degrading and losing its volume and structure [8,9,10]. FFAs contribute to the foam’s fluid drainage as well. The milk’s liquid phase separates from the foam more quickly as it decomposes. This contributes to poor foam stability by producing an unstable, watery foam with minimal structural integrity [8,9,10]. Foam stabilization requires milk proteins, particularly whey proteins like β-lactoglobulin and α-lactalbumin. By interacting with these proteins and changing their capacity to stabilize air bubbles, FFAs reduce their effectiveness. According to Ho [8], this protein–fat interaction reduces foam stability and speeds up foam collapse.

Several antimicrobial compounds are produced by protective cultures, particularly by lactic acid bacteria strains like *Lactobacillus*, *Lactococcus*, and *Pediococcus*, which prevent the growth of psychrotrophic bacteria. Among these antimicrobial agents are proteinaceous toxins called bacteriocins, which selectively target and impede the growth of bacteria that compete with them, such as psychrotrophic bacteria [11]. The food business uses a variety of bacteriocins derived from lactic acid bacteria, including *bulgarican*, *helventicin*, *plantaricin*, *nisin*, *diplococcin*, and *acidochilin*. The food sector can employ pediocins or bacteriocins made from *Pediococcus* spp. as an alternative to preservatives [12].

Hydrolytic enzymes produced by psychrotrophic bacteria during cold storage are causing technological defects in the dairy sector [13]. Milk’s triglycerides are broken down by lipases produced by psychrotrophic bacteria. Psychrotrophic bacterial counts above 1.3 × 105 Log (5.11) CFU/mL are enough to cause raw milk quality defects during transportation or storage [14,15]. The high count of microorganisms in raw milk, especially the cold-loving bacteria during transportation and storage before the heat treatment, are responsible for the formation of free fatty acids, a phenomenon which is directly related to a reduction in foam capacity and stability. Therefore, our hypothesis is to inhibit the growth of microorganisms in raw milk by natural preserves, which reduce the release of free fatty acids in milk and improve the quality of foaming properties, as well as enhancing the flavor by inhibiting the bacteria, which are responsible for proteolysis and producing peptides with bitterness.

## 2. Materials and Methods

### 2.1. Materials

Raw milk samples were collected from several Egyptian dairy farms at Shobrakhet, Nobaryia (Behira), Kafer Elzayat, Qotor (Gharbia), Domiata, and Borg Elarab. The *Pediococcus acidilactici* Ras-03 used in this study was previously isolated from Ras cheese, identified, and characterized by Elsaadany et al. [16]. The strain was from a bacteriocin producer.

Nisin (NISINPROQ) was obtained from Parroquia de Rois, D2, Polígono Industrial de Bergondo, 15165 Bergondo, La Coruña (Spain). NISINPROQ is a natural additive obtained by the fermentation of a strain of *Lactococcus lactis* subsp. lactis (GMO free).

### 2.2. Evaluation of Free Fatty Acids in Raw Milk

The free fatty acids were measured in raw and heat-treated milk samples using the method of ISO 1740 [17] as the quantity of oleic acid grams per 100 g of fat, which is equivalent to the percentage of free fatty acids.

### 2.3. Milk Treatment by CFS

Cell-free supernatant (CFS): after the strains were cultured using M17 broth media at 37 °C for 24 h, the solution was centrifuged using sigma 3-13KL centrifuge, Osterode am Harz, Germany (9500 g/10 min/4 °C) to produce the CFS [16].

Raw cow milk was sourced from a Borg Elarab, Alexandria, Egypt, dairy farm. The milk was divided into four portions. A control was used, and the CFS was added to the milk at three different concentrations: 5 mL/L, 10 mL/L, and 15 mL/L. The raw milk treatments were stored at 5 °C ± 1. Immediately following the treatment, each sample was examined for microbiological, free fatty acid, and foaming characteristics. Raw milk was stored for five days before being heat-treated for 30 s at 75 °C and cooled to 5 °C for each treatment. Samples of heat-treated milk were stored at 5 °C ± 1 for 14 days and subjected to microbiological analysis, free fatty acid, pH, flavor, and foaming characteristic assessments immediately after the heat treatment and at 2-day intervals. For flavor and pH analyses, one more treatment was carried out by adding nisin at a level of 6 mg/L.

### 2.4. Flavor Potential of Milk

Raw milk was pasteurized (75 °C/30 s) and cooled to 10 °C. Twenty well-trained panelists, including post-graduate students and staff members, assessed the milk for organoleptic characteristics (color, taste, smell) and overall acceptability using a nine-point hedonic scale with excellent (score = 9) and extremely poor (score = 0) scores. Additionally, the panelists were asked to detect any flavor defects.

### 2.5. Measuring the Foaming Capacity

Foaming of the milk by steam injection was carried out using a steam injection coffeemaker, as described by Ho et al. [2]. One hundred milliliters of sample (5 °C ± 1) was poured into a 250 mL plastic jug. The steam injection process was halted when the temperature of the sample reached about 65 °C. The samples’ foamability was measured in mL/100 mL. The graduated plastic jug was used to measure the foam volume immediately after steam injection. The entire height of the foamed sample and the liquid layer were measured as soon as foaming was complete to calculate the volume of the foam. These two values were subtracted to determine the foam volume. Foam stability in this study was expressed as the foam volume reduction after 3 and 10 min at 25 °C.

Foam capacity of 100 mL milk = [(height of foam level (mL) − height of liquid layer (mL)]

### 2.6. Physicochemical Analysis of Milk

The pH was measured using a glass electrode (Adwa model AD 1030). Total protein content was measured using the micro-Kjeldahl method, according to AOAC [18], and fat content was measured by the Gerber method, according to AOAC [18].

### 2.7. Aerobic Total Count Microorganisms

The total aerobic count of microorganisms was performed at 30 °C by the pour plate technique according to ISO 4833-1 [19].

#### 2.7.1. Counting of Psychrotrophic Microorganisms

The psychrotrophic microorganisms were enumerated at 6 ± 1 °C by the pour plate technique according to ISO 17410 [20].

#### 2.7.2. Counting the Spore Forming Bacteria

The mesophilic spore-forming bacteria counts were detected in raw milk according to Kent et al. [21].

### 2.8. Statistical Analysis

The experiment was conducted using a completely randomized design (CRD) with three replications of each treatment. The analysis of variance (ANOVA) was used to statistically evaluate all of the data. A post-hoc analysis was performed to compare means (*p* < 0.05) using Tukey’s honestly significant difference (HSD) test. The results were analyzed using the statistical technique of principle component analysis (PCA).

## 3. Results and Discussion

### 3.1. Physicochemical, Microbiological, and Flavor Analysis of Raw Milk

Samples of raw milk were collected from the bulk tanks of 15 farms in the delta region of Egypt. The samples were collected just as they were being uploaded onto trucks for delivery to dairy industries. The samples were kept in an ice box and transferred to the laboratory for analysis. The fat ranged from 3.39 to 3.81%, and the protein ranged from 3.14 to 3.37% (Table 1). The fat and protein were in the range of normal cow’s milk, and the pH was in the range of 6.72 to 6.78. The aerobic microorganisms (30 °C) were in the range of 4.34 to 5.54 log CFC/mL, the psychrotrophic microorganisms (7 °C) were in the range of 3.7 to 4.67, and the mesophilic spore-forming bacteria were in the range of 2.15 to 2.74 log CFC/mL. The aerobic bacterial count exceeded the limit of GA raw milk (the total microorganism count should not be more than 100,000 CFU/mL in GA raw cow milk, according to Egyptian standards) in six samples, which had a bacterial count greater than 100,000 CFC/mL (log 5 CFU/mL).

There was a variation among the samples in psychrotrophic microorganism counts, but most of the samples with a high aerobic microbial count also had a high level of psychrotrophic microorganisms. The variations among the samples in relation to mesophilic spore-forming counts were also related to the aerobic microorganism count in the samples. The data in Table 1 show that there was no significant relationship between microbiological quality (total microorganism count, psychrotrophic microorganism count, and mesophilic spore-forming bacteria count) and the level of free fatty acids. This suggests that the free fatty acids in fresh raw milk are mainly related to milk lipase.

The flavor scores of the milk (it was heat-treated in a water bath before tasting) were also affected by the microorganism count, as samples with a low microorganism count received high scores for flavor. The flavor of raw milk is greatly influenced by its aerobic microorganism count, which is a crucial measure of the milk’s microbiological quality. While lower levels often assist in preserving a fresher taste, high aerobic microorganism counts might cause unfavorable changes in milk quality. Good hygiene practices and fresh, clean-tasting raw milk are often indicated by a low aerobic bacterial count (below 10,000 CFU/mL). Better storage conditions, contaminated equipment, or a high aerobic microorganism count (more than 100,000 CFU/mL) can all contribute to spoiling and flavor degradation [22].

The growth of psychrotrophic microorganisms at refrigeration temperatures, even after pasteurization, poses a significant threat. Even in pasteurized milk, they generate heat-resistant proteases and lipases that do not lose their activity even after the heat treatment. When raw milk is stored in cold storage, putridity, rancidity, and bitterness can all be tastes caused by psychrotrophic spoiling [23].

### 3.2. Effect of the Physicochemical Properties and Free Fatty Acids of Raw Milk from Various Farms on the Foaming Properties

All samples were evaluated for physicochemical, microbiological, and free fatty acid (FFA) properties and foaming capacity and stability for 3 and 10 min. The FFAs/fat% ranged from 2.94 to 10.06% (Table 2). There was no relationship between the FFAs and the fat percentage. There was a significant negative (*p* ≤ 0.05) relationship between FFAs and foaming capacity, as the foaming capacity was dramatically reduced with elevated FFAs in bulk milk (Table 2).

It is common knowledge that surface activity is one of the most important properties of proteins. While the formation of emulsions and foams depends on lowering the surface tension of the phase, this drop does not account for the stability of the films. Proteins enable fluid interfaces to tolerate tangential forces from adjacent flowing liquids [24]. This has led to a great deal of research being conducted on the surface rheological properties of the protein layers that have been adsorbed. Whey proteins and caseins, for instance, do not react similarly and generate separate films. Additionally, low-molecular-weight surfactants and proteins behave differently [25].

Although milk undergoes many types of lipolysis, the consequences of elevated FFA levels are cumulative. The physiological condition and diet of the cow affect spontaneous lipolysis, which is catalyzed by lipoprotein lipases (LPL) [26,27]. Bacterial lipolysis is influenced by sanitation during milk collection and the cooling process [28,29]. Physical stress on the milk fat globule membrane causes induced lipolysis, which is influenced by parameters related to milk handling and transportation [27]. An indigenous milk enzyme called lipoprotein lipase cleaves triacyl-glyceride (TAG) to release fatty acids from the glycerol molecule, hence catalyzing spontaneous lipolysis [28]. The mammary gland produces it, and it plays a crucial role in the absorption of blood lipids to create milk fat [27,29].

Normal milk contains a high level of LPL, but it cannot reach the TAG because of a protective milk fat globule membrane [27,28,30]. Mechanical stress or agitation can reveal hydrophobic reactions in the milk fat globule membrane, where whey proteins and casein micelles can adsorb [30]. Since LPL and the casein micelle are most frequently linked, casein adsorption moves LPL closer to the TAG and increases the possibility that it may cleave to create FFAs [27,30]. Bacterial lipolysis is a similar process; however, instead of LPL catalyzing the breakdown of milk fat, the bacterial lipases do so [30]. In induced lipolysis, the TAG is exposed to LPL or bacterial lipases due to physical or chemical disruption of the milk fat globule membrane itself, which facilitates their targeting [26,27,30]. Numerous factors that cause spontaneous, bacterial, and induced lipolysis contribute to the daily variance in the amount of FFAs in bulk tank milk [28].

Therefore, the free fatty acids in raw milk can be influenced by some factors; these fall into three general categories: milk harvest variables (equipment, milk transfer, storage), herd management factors (feed), as dry feeding increases FFA levels, and cow factors (genetics, parity, lactation stage), as FFAs increased at the end of the lactation period [27,30]. It is more difficult to incorporate air into milk when there are more FFAs present. When there are high amounts of free fatty acids present, air bubbles, which are essential for foam, do not form effectively. This restricts how much foam raw milk with high FFA levels can produce. Once created, the foam structure can be severely destabilized by FFAs. The protein coatings that hold the air bubbles in the foam in place are weakened by FFAs’ propensity to move to the air–liquid interface. Poor foam stability results from the dissolution of air bubbles, a phenomenon which causes the foam to collapse more quickly. As surfactants, FFAs reduce the surface tension at the air–liquid interface, weakening the stability of the bubbles. The foam collapses quickly as a result of the bubbles coalescing (merging) due to the decreased surface tension [8,9,10].

### 3.3. Effect of the Cell-Free Supernatant of Wild Pediococcus acidilactici on the Bacterial Count of Raw and Pasteurized Milk

**Figure 1:**
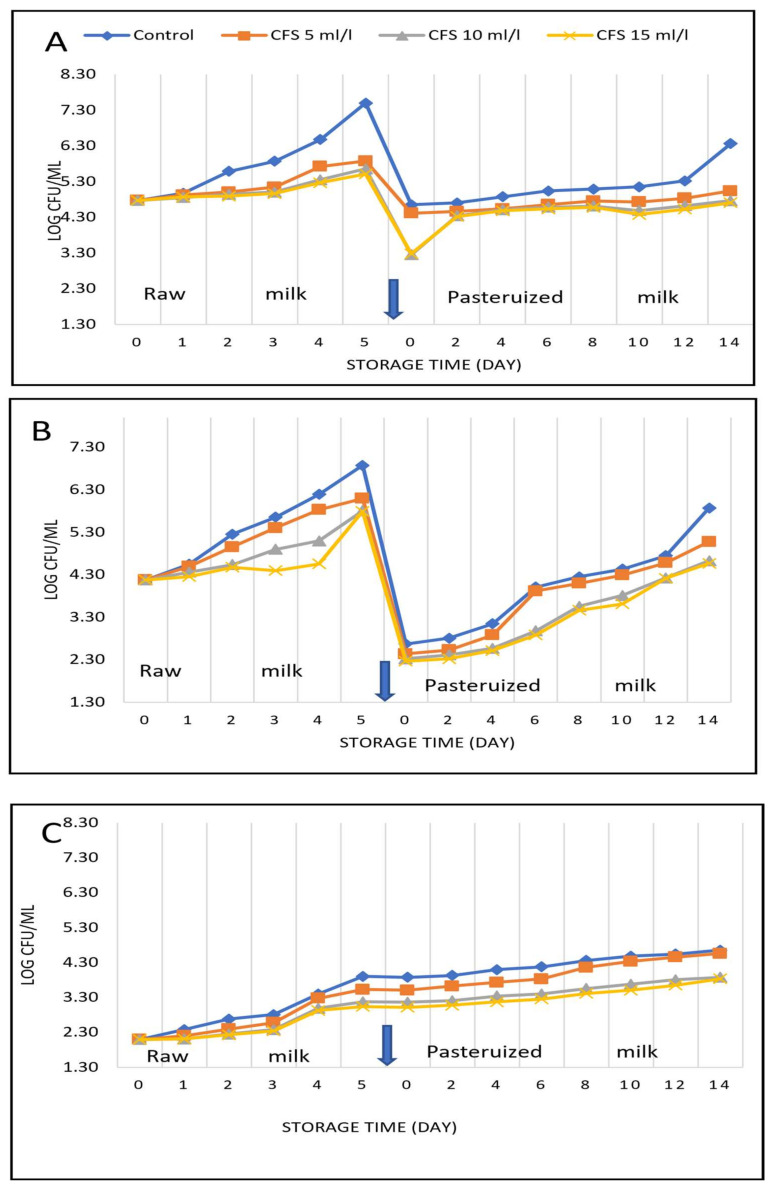
Effect of the cell-free supernatant of wild *Pediococcus acidilactici* on the microbiological quality of raw and then heat-treated milk. (**A**) Total count of microorganisms, (**B**) psychrotrophic microorganisms and, (**C**) spore-forming bacteria.

The results in Figure 1A show that the total count of aerobic microorganisms increased in raw milk stored at 5 °C ± 1 during the storage period up to 5 days, from log 4.98 to log 5.59 on day 2 and to 7.51 on day 5. A significant decrease in the count of microorganisms was observed on day 5, from log 7.51 in the control raw milk to log 5.51 in the raw milk with the highest dose of the CFS. As total count of aerobic microorganisms increased during storage, the same trend was also recorded for psychrotrophic microorganisms, as the count increased from log 4.18 to log 6.88 after 5 days, while the count of psychrotrophic microorganisms was log 5.79 in the presence of 15 mL/l CFS. Yuan et al. [31] reported that the range of psychrotrophic bacteria was 3.81 to 4.27 log CFU/mL. The 16 samples’ mean was 4.1 log CFU/m, twelve of the sixteen samples exhibited counts higher than 4 log CFU/mL, while just four samples had values lower than log 4. Following two days of storage, the psychrotrophic bacteria increased to a high of 4.9 log CFU/mL.

After day 5, the milk from all treatments was pasteurized, and there was a significant decrease in the total count of microorganisms. The total count of microorganisms increased during the storage period of pasteurized milk, but the rate of increase in the count was low in the treated milk, with the lowest being in the milk treated with the highest dose of the CFS. Therefore, the reduction in the microorganism count in the samples treated with the CFS is related to the effect of bacteriocins produced by the selected wild lactic acid bacteria in the CFS. This finding confirms previous publications [11,12].

The data in Table 3 and Figure 1 indicate a clear correlation between the bacterial count and the free fatty acids. In fresh raw milk, the free fatty acids were 0.09% as oleic acid; after five days, they increased to 0.47% in the control milk but only to 0.27% in the milk treated with the CFS at a concentration of 15 milliliters per liter. During storage, after the heat treatment, the level of FFAs increased at a low rate, but it was still high in the control raw milk, increasing by 1.21 times in 14 days. FFA levels were significantly low in the milk treated with the CFS; the treatment with the highest CFS level had the lowest FFA level.

### 3.4. Effect of the Cell-Free Supernatant of Wild Pediococcus acidilactici on the Foaming Capacity and Stability of Raw and Then Heat-Treated Milk

The results confirm that there was an increase in the total count of aerobic microorganisms, psychrotrophic microorganisms, and aerobic spore-forming bacteria. The microbial content increased when the raw milk was stored at 5 °C ± 1 for up to 5 days. Adding the cell-free supernatant (CFS) of bacteria-producing bacteriocins decreased the microbial count, and there was a significant difference when the CFS increased (Figure 1). Data in Figure 1A–C illustrate that the total count of microorganisms in the control raw milk was highest on the 5th day of storage, while this increase was lowest in the raw milk that contained the cell-free supernatant. This trend was noticed in the treatment with 15 mL/L of the CFS. As well as in pasteurized milk samples, the control pasteurized milk had the highest count of total microorganism and of psychrotrophic and spore-forming bacteria compared with samples containing the CFS.

The data in Table 4 illustrate the effect of the cell-free supernatant of wild *Pediococcus acidilactici* on the foaming capacity and stability of raw and then heat-treated milk. The results obtained confirm that the foam capacity and stability are greatly affected by the storage period of raw milk, as the rate of foam capacity and stability decreases as the storage of raw milk increases, and it was found that this is related to the increase in the rate of formation of free fatty acids. After adding the CFS, the rate of foam capacity slightly improved and the percentage of free fatty acids decreased (Table 3 and Table 4). The foam capacity and stability were enhanced by reducing the rate of free fatty acids produced by the bacterial count. The CFS inhibited the growth of microorganisms and of psychrotrophic and spore-forming bacteria, those related indirectly to enhancing the foam capacity.

The flavor quality of raw milk is mostly dependent on the aerobic microorganism count. Fresh, clean flavors are produced by low microorganism counts, while rancidity, sourness, bitterness, and even rotten overtones can emerge from high counts. The natural flavor of raw milk can be maintained for extended periods by managing the microbiological load through appropriate hygiene and storage procedures, guaranteeing a higher-quality product for consumption or further processing into dairy products [32].

Psychrotrophic microorganisms are well known for their production of lipases and proteases, both of which break down the proteins and fat in milk and cause spoiling. Through the development of protease inhibitors that prevent enzymatic breakdowns or by lowering the population of psychrotrophic microorganisms, protective cultures can indirectly restrict the production and activity of these spoilage enzymes [33].

The psychrotrophic bacterial load reduces along with the level of lipases and proteases produced, which are spoiling enzymes. The result is a slower rate of lipolysis, or the breakdown of fat, and proteolysis, or the breakdown of proteins, both of which give milk its off-textures and rancid tastes [34]. Milk and dairy products can have their shelf lives prolonged by protective cultures that inhibit the growth of psychrotrophic microorganisms. Even in cold storage, psychrotrophic microorganisms can cause spoiling since they thrive at temperatures below 7 °C which are ideal for refrigeration. Protective cultures, on the other hand, delay or stop the deterioration processes that psychrotrophs start, preserving the freshness of milk for longer [35].

In milk, bacteriocin could work as a preservative because it breaks down the cell walls of psychrotrophic bacteria and stops the production of spoiling enzymes. It can prolong the shelf life of milk by collaborating with other preservation methods, even though its direct impact on Gram-negative psychrotrophs is minimal. This makes bacteriocin an important tool for preserving the quality of milk, particularly in cold storage where psychrotrophic bacteria are a big problem [36].

Enhancing the foam capacity and foam stability of milk, two critical components in the quality of dairy products such as cappuccinos, lattes, and foamed milk beverages can be achieved with the addition of nisin and protective microorganisms. The microbiological quality of milk is predominantly influenced by both nisin and protective cultures, and this can have an indirect effect on the physical aspects of the milk, such as its foaming qualities [37].

### 3.5. Effect of the Cell-Free Supernatant of Wild Pediococcus acidilactici and Nisin on the pH and Flavour of Raw and Then Heat-Treated Milk

The data in Table 5 show that adding the CFS to raw milk affected its pH; adding the CFS caused the pH to drop down, but adding nisin at a concentration of 6 mg/L had no effect. This is as a result of fermentation acidifying the CFS. During storage, the pH decreased in all treatments, but the rate of decrease was higher in the control milk and was very low in the nisin-added treatment, which was followed by the addition of the CFS at a level of 10/1. After 14 days of storage, the pH of the milk treated with the CFS (15 mL/L) and nisin (6 mg/L) was 6.45 and 6.65, respectively, but the pH of the control milk was 5.14.

This suggests that nisin and the CFS directly affect milk quality. The flavor was also directly influenced by the addition of the CFS, with samples having the CFS receiving a low score for the flavor. Panelists detected the unclear taste of the milk after adding the CFS, but they did not notice any flavor change when nisin was added at a dosage of 6 mg/L. The flavor score gradually decreased during the storage of the control milk and was unaccepted, and bitterness was detected after 8 days.

The shelf life of the milk with the addition of the CFS at 10 and 15 milliliters per liter, respectively, was extented to 12 and 14 days respectively. The sample that had the maximum acceptable flavor and contained nisin at a level of 6 mg/L did not exhibit any bitterness. Nisin is a highly effective milk preservative because it inhibits the synthesis of spoiling enzymes and breaks down the cell walls of Gram-positive psychrotrophic bacteria [11,12]. When combined with other preservation techniques, it can increase the shelf life of milk even though it has no direct effect on Gram-negative psychrotrophs. Nisin is a helpful method for maintaining the quality of milk because psychrotrophic bacteria are a major concern during cold storage [11,12]. The addition of nisin and protective microorganisms can significantly improve the foam capacity and foam stability of milk, both of which are essential elements of the quality of dairy products like cappuccinos, lattes, and foamed milk beverages. Both nisin and protective cultures primarily affect the milk’s microbiological quality, which can alter its physical characteristics, including its ability to foam.

## 4. Conclusions

The production of milk with a high foam capacity and stability for specific purposes, particularly the making of cappuccinos and related beverages, is currently one of the most significant technological uses. The study’s findings support that the amount of free fatty acids in milk and its foam capacity are closely and inversely related. There was no significant correlation between the microbial content and the free fatty acids in some samples of raw milk, even though some raw milk samples with high fatty acid levels also had a high microbial content. This could be because raw milk has a high lipase enzyme content and a low microbial count. The addition of the cell-free supernatant of the wild *Pediococcus acidilactici* decreased microbial activity. This study advises quickly pasteurizing raw milk at the recommended temperature and time (75 °C for 30 s) for foam liquid milk to suppress the activity of the lipase enzyme and fat-degrading microorganisms and decrease the level of free fatty acids produced by some microorganisms which enhances the foam capacity rate. Additionally, adding the cell-free supernatant “bacteriocin” improves the flavor by lowering bacterial activity in milk and reducing bitterness. Since the flavour was affected by adding the CFS but there was a significant reduction in psychrotrophic microorganisms which enhances the foaming properties, further work is planned to produce the bacteriocin of wild *Pediococcus acidilactici* for application in the food industry.

## Figures and Tables

**Table 1:** Physicochemical, microbiological quality, and flavor of raw milk from individual dairy farms.

Farm	Fat %	Protein %	pH	FFA%	TC_(log CFC/mL)_	PS_(log CFU/mL)_	SF_(log CFU/mL)_	Flavor (9)
Farm 1	3.74 ^a^	3.35 ^a^	6.75	0.11 ^f^	4.34 ^c^	3.70 ^c^	2.18 ^c^	8.5 ^a^
Farm 2	3.56 ^c^	3.21 ^b^	6.72	0.16 ^cd^	4.66 ^bc^	3.93 ^c^	2.40 ^b^	8.4 ^a^
Farm 3	3.45 ^d^	3.14 ^c^	6.76	0.24 ^b^	4.55 ^c^	3.87 ^c^	2.41 ^b^	8.2 ^a^
Farm 4	3.71 ^ab^	3.30 ^a^	6.72	0.12 ^f^	4.37 ^c^	3.72 ^c^	2.15 ^c^	8.1 ^a^
Farm 5	3.65 ^c^	3.32 ^a^	6.73	0.11 ^f^	4.81 ^b^	4.01 ^c^	2.38 ^b^	8.3 ^a^
Farm 6	3.56 ^c^	3.21 ^b^	6.76	0.25 ^b^	5.18 ^a^	4.38 ^a^	2.61 ^a^	7.2 ^b^
Farm 7	3.48 ^d^	3.23 ^b^	6.74	0.35 ^a^	5.35 ^a^	4.67 ^a^	2.74 ^a^	7.4 ^b^
Farm 8	3.39 ^d^	3.34 ^a^	6.77	0.24 ^b^	5.11 ^a^	4.26 ^a^	2.66 ^a^	7.1 ^c^
Farm 9	3.62 ^c^	3.29 ^a^	6.75	0.19 ^c^	5.28 ^a^	4.42 ^a^	2.71 ^a^	7.2 ^c^
Farm 10	3.59 ^c^	3.36 ^a^	6.76	0.19 ^c^	5.10 ^a^	4.26 ^a^	2.51 ^a^	7.8 ^b^
Farm 11	3.75 ^a^	3.37 ^a^	6.75	0.11 ^f^	4.44 ^c^	3.79 ^c^	2.34 ^b^	8.5 ^a^
Farm 12	3.58 ^c^	3.26 ^ab^	6.75	0.13 ^e^	4.88 ^b^	4.06 ^b^	2.45 ^a^	8.4 ^a^
Farm 13	3.67 ^b^	3.28 ^a^	6.74	0.14 ^d^	5.54 ^a^	4.16 ^b^	2.51 ^a^	8.2 ^a^
Farm 14	3.81 ^a^	3.32 ^a^	6.78	0.16 ^cd^	4.72 ^b^	4.21 ^ab^	2.45 ^a^	8.2 ^a^
Farm 15	3.66 ^b^	3.27 ^a^	6.77	0.17 ^cd^	4.83 ^b^	4.37 ^a^	2.54 ^a^	8.1 ^a^

FFA%: free fatty acids % as oleic acid; TC: total count of microorganisms, PS: psychrotrophic microorganisms, SF: spore-forming bacteria. Means within the same column with different superscript letters ^(a–f)^ are significantly different (*p ≤* 0.05).

**Table 2:** Effect of the free fatty acids of raw milk from various farms on the foaming properties.

Farm	FFA/Fat %	Foam Capacity (mL/100 mL)		
		0	3 min	10 min
Farm 1	2.94 ^h^	39 ^a^	25 ^c^	15 ^b^
Farm 2	4.49 ^d^	29.5 ^d^	22 ^d^	11 ^c^
Farm 3	6.96 ^b^	28.5 ^d^	26 ^c^	13 ^c^
Farm 4	3.23 ^g^	33.4 ^c^	30.2 ^b^	15 ^b^
Farm 5	3.01 ^gh^	36.6 ^b^	34.9 ^a^	23 ^a^
Farm 6	7.02 ^b^	18 ^f^	8.5 ^f^	4.1 ^e^
Farm 7	10.06 ^a^	16 ^f^	5.7 ^g^	3.2 ^e^
Farm 8	7.08 ^b^	23 ^e^	18 ^e^	6.7 ^d^
Farm 9	5.25 ^c^	32 ^d^	20 ^d^	15.8 ^b^
Farm 10	5.29 ^c^	38 ^a^	34 ^a^	24 ^a^
Farm 11	2.93 ^h^	38 ^a^	36 ^a^	25 ^a^
Farm 12	3.63 ^f^	31.5 ^d^	28.7 ^b^	14.6 ^b^
Farm 13	3.81 ^f^	32.6 ^d^	29.8 ^b^	14.8 ^b^
Farm 14	4.20 ^e^	3 ^c^	31 ^b^	15.7 ^b^
Farm 15	4.64 ^d^	30 ^d^	24 ^c^	12.8 ^c^

FFA%: free fatty acids % as oleic acid; means within the same column with different superscript letters ^(a–h)^ are significantly different (*p ≤* 0.05).

**Table 3:** Effect of the cell-free supernatant of wild *Pediococcus acidilactici* on the free fatty acids (%) of milk fat as oleic acid of raw and then heat-treated milk.

Storage Time (Days)	Raw Milk						Heat-Treated Milk							
	0	1	2	3	4	5	0	2	4	6	8	10	12	14
Control	0.09 ^a^	0.14 ^a^	0.19 ^a^	0.27 ^a^	0.39 ^a^	0.47 ^a^	0.48 ^a^	0.52 ^a^	0.54 ^a^	0.55 ^a^	0.56 ^a^	0.57 ^a^	0.57 ^a^	0.58 ^a^
CFS 5 mL/L	0.10 ^a^	0.13 ^b^	0.15 ^b^	0.23 ^b^	0.25 ^b^	0.29 ^b^	0.38 ^b^	0.39 ^b^	0.39 ^b^	0.44 ^b^	0.45 ^b^	0.46 ^b^	0.45 ^b^	0.51 ^b^
CFS 10 mL/L	0.11 ^a^	0.12 ^b^	0.15 ^c^	0.20 ^c^	0.28 ^c^	0.30 ^c^	0.34 ^c^	0.31 ^c^	0.34 ^c^	0.35 ^c^	0.38 ^c^	0.37 ^c^	0.39 ^c^	0.44 ^c^
CFS 15 mL/L	0.11 ^a^	0.12 ^b^	0.15 ^c^	0.18 ^c^	0.21 ^d^	0.27 ^d^	0.26 ^d^	0.26 ^d^	0.32 ^c^	0.34 ^c^	0.31 ^d^	0.33 ^d^	0.33 ^d^	0.38 ^d^

Control: milk without any additives; CFS: cell-free supernatant, added at different concentrations of 5, 10, and 15 mL/mL. Means within the same column with different superscript letters ^(a–c)^ are significantly different (*p ≤* 0.05).

**Table 4:** Effect of the cell-free supernatant of wild *Pediococcus acidilactici* on the foaming capacity and stability of raw and then heat-treated milk.

**a. Raw Milk**																
**Storage Time (Days)**	**Received Milk**		**1st day**		**2nd day**		**3rd day**		**4th day**				**5th day**			
	**Foaming Capacity at**															
	**0**	**10 min**	**0**	**10 min**	**0**	**10 min**	**0**	**10 min**	**0**		**10 min**		**0**		**10 min**	
Control	35 ^a^	18 ^a^	34 ^a^	16 ^b^	31 ^b^	16 ^b^	26 ^b^	7 ^d^	26 ^b^		6 ^d^		23 ^b^		6 ^d^	
CFS 5 mL/L	35 ^a^	18 ^a^	35 ^a^	17 ^a^	32 ^a^	16 ^a^	29 ^a^	12 ^c^	26 ^a^		11 ^c^		25 ^a^		10 ^c^	
CFS 10 mL/L	35 ^a^	18 ^a^	35 ^a^	18 ^a^	33 ^a^	17 ^a^	31 ^a^	14 ^b^	30 ^a^		13 ^b^		29 ^a^		12 ^b^	
CFS 15 mL/L	35 ^a^	18 ^a^	35 ^a^	18 ^a^	33 ^a^	17 ^a^	32 ^a^	15 ^a^	31 ^a^		14 ^a^		30 ^a^		14 ^a^	
**b. After Pasteurization**																
**Storage Time (Days)**	**0**		**2**		**4**		**6**		**8**		**10**		**12**		**14**	
**Foaming Capacity at**																
	**0**	**10 min**	**0**	**10 min**	**0**	**10 min**	**0**	**10 min**	**0**	**10 min**	**0**	**10 min**	**0**	**10 min**	**0**	**10 min**
Control	23 ^d^	9 ^c^	21 ^c^	7 ^c^	18 ^c^	7 ^c^	17 ^c^	6 ^d^	17 ^c^	5 ^c^	15 ^c^	5 ^d^	14 ^c^	4 ^c^	10 ^c^	3 ^d^
CFS 5 mL/L	26 ^c^	14 ^b^	26 ^b^	12 ^b^	24 ^b^	12 ^b^	23 ^b^	11 ^c^	22 ^b^	10 ^b^	21 ^b^	9 ^c^	18 ^b^	8 ^b^	16 ^b^	7 ^c^
CFS 10 mL/L	29 ^b^	16 ^a^	29 ^a^	15 ^a^	29 ^a^	14 ^a^	27 ^b^	13 ^b^	26 ^a^	13 ^a^	26 ^b^	11 ^b^	24 ^a^	11 ^a^	22 ^b^	10 ^b^
CFS 15 mL/L	31 ^a^	17 ^a^	30 ^a^	15 ^a^	29 ^a^	15 ^a^	28 ^a^	14 ^a^	28 ^a^	14 ^a^	27 ^a^	14 ^a^	27 ^a^	13 ^a^	26 ^a^	12 ^a^

Control: milk without any additives; CFS: cell-free supernatant, added at different concentrations of 5, 10, and 15 mL/mL. Means within the same column with different superscript letters ^(a–c)^ are significantly different (*p ≤* 0.05).

**Table 5:** Effect of the cell-free supernatant of wild *Pediococcus acidilactici* and nisin on the pH and flavor of raw and then heat-treated milk.

Sample	pH					Flavor Score (Hedonic Scale 9)				
	Control	CFS 5 mL/L	CFS 10 mL/L	CFS 15 mL/L	Nisin 6 mg/L	Control	CFS 5 mL/L	CFS 10 mL/L	CFS 15 mL/L	Nisin 6 mg/L
Day 0-R	6.71	6.68	6.66	6.64	6.71	8.31 ^a^	7.37 ^b^	7.12 ^c^	6.78 ^d^	8.38 ^a^
Day 1-R	6.68	6.68	6.66	6.64	6.70	8.05 ^b^	7.35 ^c^	7.11 ^d^	6.57 ^e^	8.24 ^a^
Day 2-R	6.66	6.67	6.65	6.63	6.70	7.57 ^b^	7.31 ^b^	6.94 ^c^	6.46 ^d^	8.17 ^a^
Day 3-R	6.65	6.66	6.65	6.63	6.69	7.27 ^b^	6.64 ^c^	6.64 ^c^	6.41 ^c^	8.09 ^a^
Day 4-R	6.63	6.64	6.64	6.62	6.68	7.02 ^b^	6.54 ^c^	6.54 ^c^	6.38 ^c^	7.98 ^a^
Day 5-R	6.60	6.63	6.64	6.62	6.67	6.95 ^b^	6.45 ^c^	6.48 ^c^	6.29 ^d^	7.81 ^a^
Day 0-P	6.6	6.63	6.64	6.62	6.67	6.92 ^b^	6.41 ^c^	6.42 ^c^	6.28 ^c^	7.80 ^a^
Day 2-P	6.6	6.63	6.64	6.61	6.67	6.18 ^c^	6.40 ^b^	6.36 ^b^	6.19 ^c^	7.78 ^a^
Day 4-P	6.58	6.62	6.64	6.60	6.66	5.59 ^d^	6.36 ^b^	6.34 ^b^	6.14 ^c^	7.62 ^a^
Day 6-P	6.58	6.61	6.64	6.60	6.66	5.06 ^c^	6.29 ^b^	6.17 ^b^	6.08 ^b^	7.54 ^a^
Day 8-P	6.54	6.60	6.61	6.60	6.66	ND	6.07 ^b^	6.02 ^b^	6.06 ^b^	7.45 ^a^
Day 10-P	5.85	6.43	6.54	6.59	6.66	ND	5.89 ^c^	6.47 ^b^	6.34 ^b^	7.36 ^a^
Day 12-P	5.42	6.15	6.45	6.54	6.66	ND	ND	5.35 ^c^	6.22 ^b^	7.25 ^a^
Day 14-P	5.14	5.87	6.35	6.45	6.65	ND	ND	ND	6.14 ^b^	7.08 ^a^

Means within the same row with different superscript letters ^(a–e)^ are significantly different (*p ≤* 0.05).

## Data Availability

The original contributions presented in this study are included in the article. Further inquiries can be directed to the corresponding authors.

## References

[1] Hettiarachchi C.A., Corzo-Martínez M., Mohan M.S., Harte F.M. (2018). Enhanced Foaming and Emulsifying Properties of High-Pressure-Jet-Processed Skim Milk. Int. Dairy J..

[2] Ho T.M., Bhandari B.R., Bansal N. (2024). Foaming Properties of Milk Samples Collected at Various Processing Stages in a Dairy Processing Factory across Two Seasons. J. Sci. Food Agric..

[3] Hatakeyama S., Akiyama M., Yoneyama R., Watanabe K., Koizumi R., Miyaji K., Mizota Y., Ikeda M., Wakao S. (2019). Effects of Manufacturing Conditions on the Foaming Properties of Milk and Sensory Characteris-Tics of Foamed Milk. LWT.

[4] Heck J.M.L., Van Valenberg H.J.F., Dijkstra J., Van Hooijdonk A.C.M. (2009). Seasonal Variation in the Dutch Bovine Raw Milk Composition. J. Dairy Sci..

[5] Chen B., Lewis M.J., Grandison A.S. (2014). Effect of Seasonal Variation on the Composition and Properties of Raw Milk Destined for Processing in the UK. Food Chem..

[6] Atasoy A.F., Türkoğlu H. (2008). Changes of Composition and Free Fatty Acid Contents of Urfa Cheeses (a White-Brined Turkish Cheese) during Ripening: Effects of Heat Treatments and Starter Cultures. Food Chem..

[7] Mottar J.F. (2020). Effect on the Quality of Dairy Products.

[8] Ho T.M., Bhandari B.R., Bansal N. (2022). Functionality of Bovine Milk Proteins and Other Factors in Foaming Properties of Milk: A Review. Crit. Rev. Food Sci. Nutr..

[9] Ho T.M., Bhandari B., Bansal N. (2020). Influence of Milk Fat on Foam Formation, Foam Stability and Functionality of Aerated Dairy Products. Dairy Fat Products and Functionality: Fundamental Science and Technology.

[10] Ho T.M., Tanzil A., Bhandari B.R., Bansal N. (2023). Effect of Surfactant Type on Foaming Properties of Milk. Food Bioprocess Technol..

[11] El-Sayed A.I., El-Sayed I.M., Awad S. (2023). Bacteriocins: Nisin as an Alternative Source to Chemi-Cal Preservatives. Natural Food Preservatives.

[12] Setiarto R.H.B., Anshory L. (2024). Bacteriocın, Plantaricin and Pediocin Bıosynthesis in Lactic Acid Bacteria, Antimicrobial Mechanism and Applications as Food Preservatives. Curr. Appl. Sci. Technol..

[13] Machado S.G., Baglinière F., Marchand S., Van Coillie E., Vanetti M.C., De Block J., Heyndrickx M. (2017). The biodiversity of the microbiota producing heat-resistant enzymes responsible for spoilage in processed bovine milk and dairy products. Front. Microbiol..

[14] Marchand S., Heylen K., Messens W., Coudijzer K., De Vos P., Dewettinck K., Herman L., De Block J., Heyndrickx M. (2009). Seasonal influence on heat-resistant proteolytic capacity of Pseudomonas lundensis and Pseudomonas fragi, predominant milk spoilers isolated from Belgian raw milk samples. Environ. Microbiol..

[15] Yuan L., Sadiq F.A., Burmølle M., Wang N.I., He G. (2019). Insights into psychrotrophic bacteria in raw milk: A review. J. Food Prot..

[16] Elsaadany K., El-Sayed A.I., Awad S. (2024). Identification, Safety Assessment, and Antimicrobial Characteristics of Cocci Lactic Acid Bacteria Isolated from Traditional Egyptian Dairy Products. Foods.

[17] (2004). Microbiology of Food and Animal Feeding Stuffs—Horizontal Method for the Enumeration of Presumptive Bacillus Cereus—Olony-Count Technique.

[18] AOAC (2019). Official Methods of Analysis of the Association of Official Analytical Chemists: Official Methods of Analysis of AOAC International.

[19] (2013). Microbiology of the Food Chain—Horizontal Method for the Enumeration of Microor-Ganisms Part 1: Colony Count at 30 °C by the Pour Plate Technique.

[20] (2019). Microbiology of the Food Chain—Horizontal Method for the Enumeration of Psy-Chrotrophic Microorganisms.

[21] Kent D.J., Chauhan K., Boor K.J., Wiedmann M., Martin N.H. (2016). Spore Test Parameters Matter: Mesophilic and Thermophilic Spore Counts Detected in Raw Milk and Dairy Powders Differ Significantly by Test Method. J. Dairy Sci..

[22] Li H., Xi B., Yang X., Wang H., He X., Li W., Gao Y. (2022). Evaluation of Change in Quality Indices and Volatile Flavor Components in Raw Milk during Refrigerated Storage. LWT.

[23] Brasca M., Decimo M., Morandi S., Machado S.G., Bagliniére F., Vanetti M.C.D., Poltronieri P. (2017). Psychrotrophic Bacteria. Microbiology in Dairy Processing: Challenges and Opportunities.

[24] Lucassen-Reynders E.H., Benjamins J., Fainerman V.B. (2010). Dilational Rheology of Protein Films Adsorbed at Fluid Interfaces. Curr. Opin. Colloid Interface Sci..

[25] Xiong X., Ho M.T., Bhandari B., Bansal N. (2020). Foaming Properties of Milk Protein Dispersions at Different Protein Content and Casein to Whey Protein Ratios. Int. Dairy J..

[26] Rasmussen M.D., Wiking L., Bjerring M., Larsen H.C. (2006). Influence of Air Intake on the Concen-Tration of Free Fatty Acids and Vacuum Fluctuations during Automatic Milking. J. Dairy Sci..

[27] Wiking L., Nielsen J.H., Båvius A.K., Edvardsson A., Svennersten-Sjaunja K. (2006). Impact of Milking Frequencies on the Level of Free Fatty Acids in Milk, Fat Globule Size, and Fatty Acid Composi-Tion. J. Dairy Sci..

[28] Wiking L., Bjerring M., Løkke M.M., Løvendahl P., Kristensen T. (2019). Herd Factors Influencing Free Fatty Acid Concentrations in Bulk Tank Milk. J. Dairy Res..

[29] Zhou X., Hadiatullah H., Guo T., Yao Y., Li C., Wang X. (2021). Dairy Processing Affects the Gut Digestion and Microecology by Changing the Structure and Composition of Milk Fat Globules. J. Agric. Food Chem..

[30] Wiking L. (2005). Milk Fat Globule Stability: Lipolysis with Special Reference to Automatic Milking Systems. Swedish University of Agricultural Sciences Uppsala. Acta Univ. Agric. Sueciae.

[31] Yuan L., Sadiq F.A., Liu T., Flint S., Chen J., Yang H., Gu J., Zhang G., He G. (2017). Psychrotrophic bacterial populations in Chinese raw dairy milk. LWT.

[32] Murphy S.C., Martin N.H., Barbano D.M., Wiedmann M. (2016). Influence of Raw Milk Quality on Processed Dairy Products: How Do Raw Milk Quality Test Results Relate to Product Quality and Yield?. J. Dairy Sci..

[33] Yalew K., Pang X., Huang S., Zhang S., Yang X., Xie N., Wang Y., Lv J., Li X. (2024). Recent Development in Detection and Control of Psychrotrophic Bacteria in Dairy Production: Ensuring Milk Quali-Ty. Foods.

[34] Fusco V., Chieffi D., Fanelli F., Logrieco A.F., Cho G.S., Kabisch J., Böhnlein C., Franz C.M.A.P. (2020). Microbial Quality and Safety of Milk and Milk Products in the 21st Century. Compr. Rev. Food Sci. Food Saf..

[35] Rauh V., Xiao Y. (2022). The Shelf Life of Heat-Treated Dairy Products. Int. Dairy J..

[36] Ibrahim A., Awad S. (2018). Selection and Identification of Protective Culture for Controlling Staphylococcus Aureus in Fresh Domiati like Cheese. J. Food Saf..

[37] Silva C.C., Silva S.P., Ribeiro S.C. (2018). Application of Bacteriocins and Protective Cultures in Dairy Food Preservation. Front. Microbiol..

